# Measuring Vascular Recovery Rate After Exercise ^†^

**DOI:** 10.3390/ecsa-5-05746

**Published:** 2018-11-14

**Authors:** Halil Dijab, Jordi Alastruey, Peter H. Charlton

**Affiliations:** Department of Biomedical Engineering, School of Biomedical Engineering and Imaging Sciences, King’s College London, King’s Health Partners, St Thomas’ Hospital, London SE1 7EH, UK

**Keywords:** wearable sensors, arterial stiffness, photoplethysmogram, exercise, heart rate recovery

## Abstract

The rate at which an individual recovers from exercise is known to be indicative of cardiovascular risk. It has been widely shown that the reduction in heart rate immediately after exercise is predictive of mortality. However, little research has been conducted into whether the time taken for the blood vessels to return to normal is also indicative of risk. In this study, we present a novel approach to assess vascular recovery rate (VRR) using the photoplethysmogram (PPG) signal, which is monitored by smart wearables. The VORTAL dataset (http://peterhcharlton.github.io/RRest/) was used for this study, containing PPG signals from 39 healthy subjects before (baseline) and after exercise. 31 VRR indices were extracted from the PPG pulse wave shape, as well as heart rate for comparison. The rate at which indices returned to baseline after exercise was quantified, and the consistency of changes between subjects was assessed statistically. Many VRR indices exhibited changes after exercise which were consistent between subjects. Indices derived from the timings and second derivative of pulse waves were identified as candidates for future work. The rate at which the indices returned to baseline differed between indices and subjects, indicating that they may provide additional information beyond that of heart rate, and that they may be useful for stratifying subjects. This study demonstrated the feasibility of assessing VRR after exercise from the PPG. Future studies should investigate whether VRR indices are associated with cardiovascular fitness, and the potential utility of incorporating the indices into wearable sensors.

## 1 Introduction

The rate at which the body recovers from exercise is known to be indicative of cardiovascular risk. It has been widely shown that the rate at which the heart rate recovers immediately after exercise is predictive of cardiovascular events and all-cause mortality [1]. Indeed, heart rate recovery (the number of beats per minute by which the heart rate falls one minute after cessation of exercise) has recently been included in recommendations for cardiopulmonary exercise testing [2]. However, little research has been conducted into whether the time taken for the blood vessels to return to normal, termed the vascular recovery rate (VRR), is also indicative of risk. If the VRR could be easily measured using wearable sensors, then it could be useful for assessing health and fitness.

**Figure 1:**
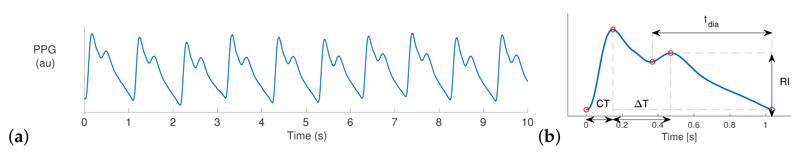
The photoplethysmogram (PPG) signal (**a**) A 10-s recording, exhibiting a pulse wave for each heart beat—approximately one per second; (**b**) Extracting four exemplary vascular recovery rate (VRR) indices from a pulse wave (defined in Abbreviations section at end). au: arbitrary units

Many smart watches and fitness bands measure the photoplethysmogram (PPG) signal [3]. The PPG is the amount of light either reflected from or transmitted through an illuminated tissue bed. Wrist-worn devices usually obtain the PPG signal by illuminating the skin at the wrist using a light-emitting diode and measuring the amount of light reflected from the skin using a photodetector. The resulting PPG signal exhibits a pulse wave for each heartbeat, as shown in Figure 1. The pulse waves are primarily caused by the expansion and relaxation of blood vessels due to the increase and decrease in blood pressure with each heartbeat. Consequently, pulse wave shape is influenced by both the ejection of blood from the heart and the mechanical properties of the blood vessels.

Previous research indicates that the stiffness of arteries changes during exercise, and these changes persist for several minutes afterwards [4]. The changes may be beneficial to health and fitness as they reduce the work required for the heart to pump blood around the body [5]. The effects of these changes on pulse wave shape have been investigated in [5,6]. However, to our knowledge no tool has been developed for analyzing PPG pulse waves to derive a measure of the VRR.

The aim of this study was to investigate the feasibility of measuring VRR indices from the PPG. Several measurements of pulse wave shape were extracted from a dataset of PPG signals acquired before and after exercise. The inter-subject consistency of changes in VRR indices during recovery from exercise, and the rate at which indices returned to baseline values, were assessed. If this approach is found to provide useful indicators of cardiovascular health, then it could be incorporated into wearable sensors for use in both everyday and clinical settings.

## 2 Methods

### 2.1 Dataset of Photoplethysmogram (PPG) Signals Acquired before and after Exercise

The VORTAL dataset contains PPG signals acquired in a controlled laboratory setting. Signals were acquired from 39 young, healthy subjects while lying down for approximately 10 min both before and after exercise, as described in [7]. The PPG signals were measured using an MLT1020FC finger clip infrared reflection plethysmograph and digitized at 500 Hz. The exercise consisted of running on a treadmill until 30 s after the heart rate (HR) reached 85% of the age-predicted maximum.

### 2.2 Extracting Vascular Recovery Rate (VRR) Indices from the PPG

VRR indices were extracted from the PPG as follows. PPG signals were band-pass filtered to eliminate very low and very high frequency content, using −3 dB cut-offs of 0.07 and 16.75 Hz. Signal quality was assessed for consecutive 10 s signal segments using the approach in [8]. Low quality segments were excluded from the analysis. Pulse waves were identified using the algorithm described in [9]. Pulse wave analysis techniques were used to extract 31 vascular stiffness indices (termed VRR indices) and HR from each PPG pulse wave, as described in [10]. These time series were median filtered to attenuate high frequency variations (using a filter of order 15 beats).

### 2.3 Identifying Significant Changes in VRR Indices

The Wilcoxon Rank Sum test was used to identify any statistically significant changes in VRR indices during recovery from exercise (at the 5% significance level). This was performed by comparing the initial and final 5% of beats in the recovery recording. Two tests were performed for each VRR: one to identify a positive change, and one to identify a negative change.

### 2.4 Quantifying Changes in VRR Indices After Exercise

Changes in VRR indices immediately after exercise were assessed as follows. The mean values of each VRR index during the initial and final 5% of beats in the recovery recording were calculated $\left({\bar{VRR}}_{i,init}\;\text{and}\;{\bar{VRR}}_{i,final}\right).$ The baseline value of each VRR index was calculated as its mean value during the recording at rest prior to exercise $\left({\bar{VRR}}_{i,base}\right).$ The percentage change in each VRR index (*VRR_i_*) per minute was calculated using (where *T* is the duration of the recovery recording in minutes):

(1) $$\text{Percentage}\;\text{change}\;\text{in}\;VRR_{i}\;\left[\%\;{\text{min}}^{-1}\right]=100\times \frac{{\bar{VRR}}_{i,final}-{\bar{VRR}}_{i,init}}{\left|{\bar{VRR}}_{i,init}-{\bar{VRR}}_{i,base}\right|}\times \frac{1}{T}.$$

## 3 Results and Discussion

### 3.1 Exemplary Changes in VRR Indices after Exercise

**Figure 2:**
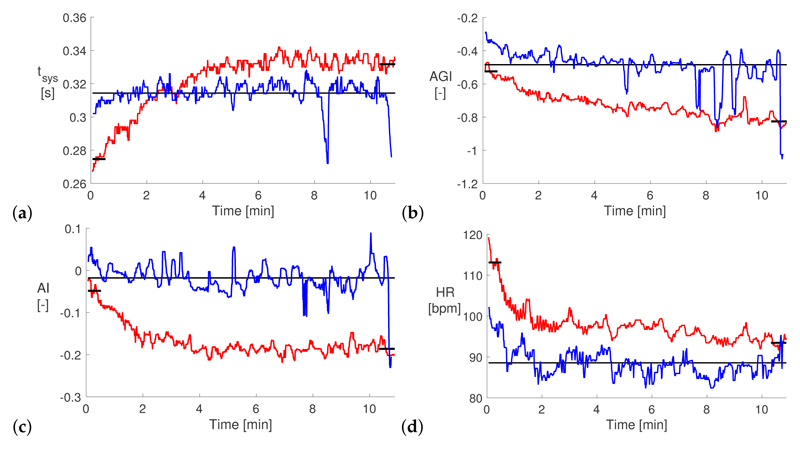
Exemplary Changes in VRR Indices After Exercise (**a**) duration of systole (*t_sys_*); (**b**) ageing index (AGI); (**c**) augmentation index (AI); (**d**) heart rate (HR). Red lines indicate the recovery recording, blue lines the rest recording prior to exercise, and black lines show the mean VRR values during the initial and final parts of the recovery recording and the baseline mean during the rest recording. bpm: beats per minute; au: arbitrary units

Figure 2 shows exemplary changes in selected VRR indices after exercise for one subject. In this example the duration of systole (*t_sys_*, shown in Figure 2a) increased over the first five minutes of the post-exercise recording and plateaued at a slightly higher value than the baseline value. In contrast, the ageing index (AGI) and augmentation index (AI) in Figure 2b,c decreased after exercise, moving further from their baseline values. Previous studies have also found that AI remains below its baseline value for 10 s to hours after exercise. Finally, the HR (shown in Figure 2d), almost returned to its baseline value during the 10 min after exercise. Most of the change in HR occurred within the first two minutes, as opposed to the five minutes for *t_sys_*. This indicates that even though both these indices are based on timings of the pulse wave, the mechanisms responsible for their changes may differ. Some high frequency variation in indices was still present, despite the use of median filtering, indicating that in the future it may be useful to adopt stricter signal quality criteria when selecting which pulse waves to include in the analysis. The VRR indices had not returned to baseline values by the end of the recording, indicating that future studies should record signals for longer than 10 min post-exercise (which is supported by [4]).

### 3.2 The Consistency of Changes in VRR Indices Between Subjects

**Figure 3:**
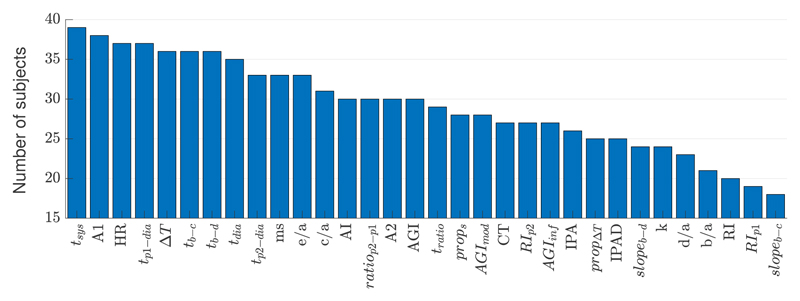
The number of subjects (out of 39) who exhibited a significant change in each VRR index in a particular direction after exercise. Indices are defined in the Abbreviations section (see end).

Figure 3 shows the number of subjects who exhibited a significant change in each VRR index in a particular direction after exercise. Several indices changed in a consistent direction for most subjects. These included indices calculated from the timings (indicated by *t*) and second derivative of the pulse wave (e.g., *e/a, c/a* and AGI). For instance, the duration of systole (*t_sys_*) increased during recovery for all 39 subjects. Similarly, the systolic pulse wave area (*A*1) and several timing measurements all increased in most subjects, and the HR decreased as observed previously [4].

### 3.3 The Rate of Changes in VRR Indices

**Figure 4:**
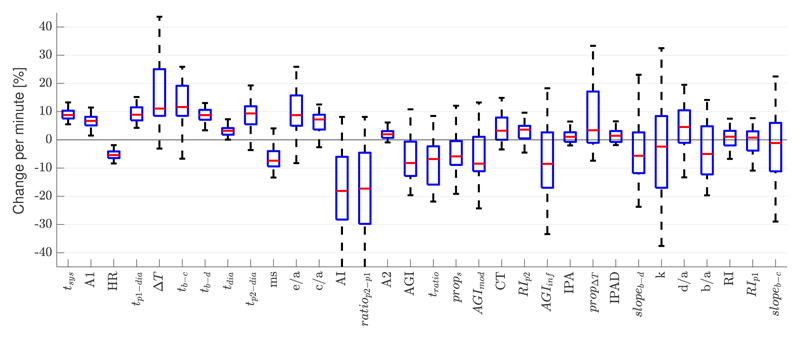
The distributions of the percentage change in each VRR index per minute during recovery from exercise. Boxplots show the median, lower, and upper quartiles.

Figure 4 shows the percentage change in VRR indices per minute during recovery from exercise. There was a wide variety in the rate at which different indices changed after exercise. This indicates that different indices may be influenced by different cardiovascular properties and may therefore contain complementary information on the state of the vasculature. In addition, the rate of change of some indices differed substantially between different subjects (e.g., Δ*T*), whereas others showed similar rates of change between subjects (e.g., *t_sys_*). This indicates that the changes in indices may be indicative of subject-specific recovery from exercise and may therefore be useful for stratifying subjects.

### 3.4 Limitations and Future Work

Firstly, the dataset used in this study was acquired from a relatively homogeneous group of healthy subjects. In the future it would be helpful to investigate whether VRR indices change differently after exercise in different groups of subjects (for instance with different levels of health or fitness). This would allow one to assess the potential utility of VRR indices for clinical decision support. Secondly, the post-exercise recordings were acquired shortly after exercise ceased (after time for subjects to move from the treadmill to the bed), and for approximately 10 min. In the future it would be helpful to study recordings from immediately after exercise, and for a longer time period to capture more of the recovery. Thirdly, future work should investigate the mechanisms underlying changes in VRR indices to determine which indices would be most suitable for assessing cardiovascular health.

### 3.5 Applications

We envisage two settings in which VRR could potentially be used: in daily life, and in exercise tests. If the approach was implemented in smart wearables (e.g., fitness bands), then individuals could measure their VRR in routine activities, such as stair climbing, walking, and running. The challenges for this application are that: (i) the activities would not be standardized, so further work would be required to contextualize the VRR according to the level of activity; and (ii) subjects may still be moving after the end of the activity, impairing PPG signal quality. The Parkrun initiative provides a convenient, relatively standardized, weekly exercise regime [11] which could allow self-assessment of VRR. If VRR is found to give clinically useful information then it may be useful to measure it in exercise tests, potentially providing additional insight into the body's ability to recover from exercise.

## 4 Conclusions

This study demonstrated the feasibility of extracting VRR indices from the PPG signal, which is routinely acquired by smart wearables. VRR indices which exhibited consistent inter-subject changes after exercise were identified as candidates for future research (namely those extracted from pulse wave timings and from its second derivative). We observed that the rates of changes in VRR indices differed between indices, and between subjects. This indicates that different indices may be influenced by different physiological mechanisms, and that the recovery may be subject-specific. Therefore, further work should investigate the physiological origins of changes in VRR indices, and determine whether they could be used to usefully assess cardiovascular health in both clinical settings and everyday life.

## Abbreviations

The following abbreviations are used to denote the VRR indices. Further explanation is provided in [10] (Table 3).

*t_sys_*: duration of systole
*A*1, *A*2: systolic, diastolic area
*HR*: heart rate
*t*_*p*1–*dia*_: time between *p*1 and *dia*
Δ*T*: time between *s* and *dia*
*t_b–c_*: time between *b* and *c*
*t_b–d_*: time between *b* and *d*
*t_dia_*: duration of diastole
*t*_*p*2–*dia*_: time between *p*2 and *dia*
*ms*: maximum slope
*e/a*: amplitude of *e* divided by that of *a*
*c/a*: amplitude of *c* divided by that of *a*
*AI*: augmentation index
*ratio*_*p*2–*p*1_: amplitude of *p*2 divided by *p*1
*AGI, AGI_mod_*: (modified) ageing index
*t_ratio_*: time of *s* divided by time of time of *dic*
*prop_s_*: time of *s* divided by pulse wave duration
*RI*_*p*1_: reflection index calculated using *p*1
*CT*: time of *s*
*RI*_*p*2_: reflection index calculated using *p*2
*AGI_inf_*: informal ageing index
*IPA*: *A*2/*A*1
*prop*_Δ*T*_: Δ*T* divided by duration of pulse wave
*IPAD*: *IPA* = *d/a*
*slope_b–d_, slope_b–c_*: slope from *b* to *d,* or *c*
*k*: spring constant
*d/a*: amplitude of *d* divided by that of *a*
*b/a*: amplitude of *b* divided by that of *a*
RI: reflection index calculated using *s*
